# Encoding the Photoreceptors of the Human Eye

**DOI:** 10.7759/cureus.30125

**Published:** 2022-10-10

**Authors:** Shreya Roy, Prachi Nagrale

**Affiliations:** 1 Medical Student, Jawaharlal Nehru Medical College, Datta Meghe Institute of Medical Sciences, Wardha, IND; 2 Ophthalmology, Jawaharlal Nehru Medical College, Datta Meghe Institute of Medical Sciences, Wardha, IND

**Keywords:** visual aid, electroretinography, neural networks, phototransduction, photoreceptors

## Abstract

This review aims to assess the anatomy of the human eye with a focus on exploring opportunities to mimic certain functionalities of photoreceptors in the optical system. This can help restore vision issues in people who had normal vision earlier, but their vision was impaired due to reasons that damaged parts of the eye; however, the functionality of the optic nerve remained intact. It is a conceptual article where the methodology to simulate artificial photoreceptors is discussed.

## Introduction and background

The human eye is a complicated yet efficient organ that helps us visualize our surroundings. It helps us see and interpret colors and objects of various shapes and sizes, with great accuracy and precision. It has also played an important role in the survival and evolution of our species, thus forming an integral organ system in the human body [1].

Light falling on an object is reflected by it and falls on the retina of the eye resulting in vision or sight. This process seems simple theoretically but is realized by a series of complex assemblies of organs that help to transform the photons (light particles) into electrical signals that the brain interprets [1-4]. If we broadly categorize the functions of the optic system, we see four phases during this transition of photons that ultimately leads to vision (or sight). First, the lens needs to direct the light from an image to fall on the retina; second, it needs to be converted into an electrical signal by photoreceptors [1-4]. Next, the optic nerve transfers these electrical signals to our brain, and, finally, the brain needs to interpret these signals and associate them with the image we see [1-4]. While the signals go through the transition across various parts of the system, the mystery deepens as each subsequent part gets more complex and performs a task with higher complexity.

Medical science has made several attempts to isolate the functionalities of this entire system and decode them step by step. It is understood that the image needs to form on the retina for normal vision [1]. Visual impairment due to the loss of accommodating power of the lens has been already rectified. We have a wide variety and quality of lenses that can correct such issues [5]. Unlike spectacles, modern-day contact lenses have also been able to keep the aesthetics of an individual, where one cannot even identify the difference [6].

Having ensured that the image now forms on the retina [1], the next problem at hand is impaired vision due to the lack of function of photoreceptors. In the normal eye, a complex set of biochemical steps collectively known as phototransduction ensures the conversion of photons to an electric signal [2-4,7]. The theory proposed is to abstract the complexities of this process and stimulate this with convolutional neural networks. For this, there is a need to first interpret the photonic signals, decode them, and encode them back to generate electrical signals identical to what phototransduction would have done and pass it to the next step [8].

Replicating the functioning of the human eye by encoding the photoreceptors present in it into a device can serve the purpose of visual aid in individuals with visual impairment due to photoreceptor issues [9]. If modern-day human technological advancements succeed in replicating the human eye, it could be a revolutionary change in health care as it will help reduce the prevalence of visual impairment in human society, thus reducing the burden of disease [10].

## Review

Phototransduction

**Figure 1:**
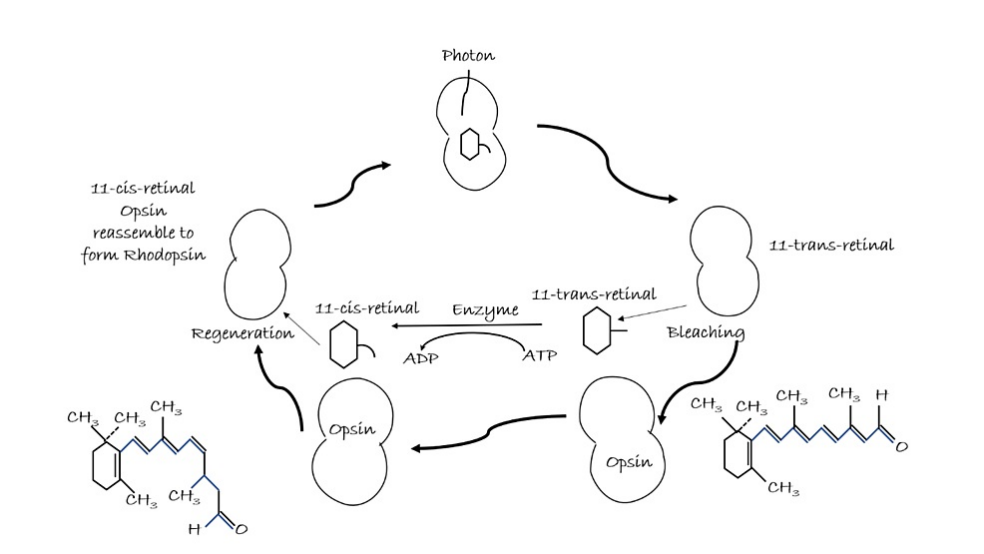
Phototransduction. Authors’ own creation [3,4,7,11].

The eyes have photoreceptor cells in the retina, namely, rods and cones [1-4]. Rods and cones transform light (electromagnetic signals) into electrical signals [2-4]. There are 6 × 106 cones and 100 × 106 rods in our retina [11]. Photoreceptor proteins called opsin combine with retinal to form rhodopsin in rods and iodopsin in cones [2,3,11-14]. Because cones have three different types of opsins that enable them to be sensitive to different wavelengths of colored light, they are classified into S cones, M cones, and L cones depending on their sensitivity to light waves of small, medium, and long wavelength, respectively [15,16]. The retinal exists as two isomers, viz. cis-form and trans-form [12,13,17]. While in dark, it exists in cis-form, light stimulation triggers the isomerization to trans-form [12,13,17,18]. Light stimulation triggers the photoreceptors to release fewer neurotransmitters which depolarize or hyperpolarize the bipolar cells. The non-stimulated photoreceptors are depolarized; in the case of light stimulation, these photoreceptors become hyperpolarized [17-19]. The mechanism of depolarization and hyperpolarization occurs with the help of calcium ions and nucleotide cyclic guanosine monophosphate. The photoreceptors are usually depolarized due to the increased number of calcium ion channels that are open in the synaptic terminal in dark. This results in the increased rate of release of the transmitter in the synaptic terminals [7,19]. On the other hand, photoreceptors are hyperpolarized due to a decrease in the number of calcium ion channels that are open which results in a decreased rate of release of the transmitter in the synaptic terminals during light hours [7,19]. Increased levels of cyclic guanosine monophosphate are responsible for keeping the ion channels open during the dark. There is a significant drop in the cyclic guanosine monophosphate levels due to light which results in hyperpolarization leading to decreased rate of release of the transmitter at the synaptic level in the photoreceptors [7,19]. A suitable stimulus causes the activation of receptors through membrane depolarization, which results in an action potential in other sensory systems. However, in the retina of the human eye, the process of change in the rate of transmitter release onto the postsynaptic neuron occurs as a result of graded potential [7,19]. Figure 1 diagrammatically explains the steps included in phototransduction or the visual cycle.

Convolutional neural network

In a convolutional neural network, there are three basic layers, of which two are on the periphery, the input layer and the output layer. The third layer is hidden and can have multiple layers within. In a convolutional neural network, each hidden layer convolves the inputs and forwards them to the next hidden layer [20,21]. A hidden layer receives input from a restricted area of the layer before. As convolution is applied in each subsequent layer, input is received from a larger area of the previous layer compared to the layer before [22]. In the case of multiple layers of a convolutional neural network, the first layer aims to figure out the low-level characteristics of an image such as edges, color, gradient, orientation, etc. [23]. With additional layers, even high-level characteristics are identified. This network then starts to behave closer to humans [21]. In the case of multiple hidden layers, the convolutional layers are followed by activation layers and, thereafter, pooling layers [24].

The convolutional neural network concept is analogous to the nerve cells in the human brain. A biological neuron receives multiple signals through the synapses contacting its dendrites and sends a single stream of action potentials out through its axon. The conversion of a complex pattern of inputs into a simple decision led to the theory that each neuron reduces complexity by categorizing its input patterns. Artificial neural network models are composed of units that combine multiple inputs and produce a single output. They stem from the way the visual cortex is organized [25]. Nerve cells respond to any stimuli in a restricted region of the visual field [26]. This is the receptive field [26]. The visual area is covered by the collection of receptive fields [26].

**Figure 2:**
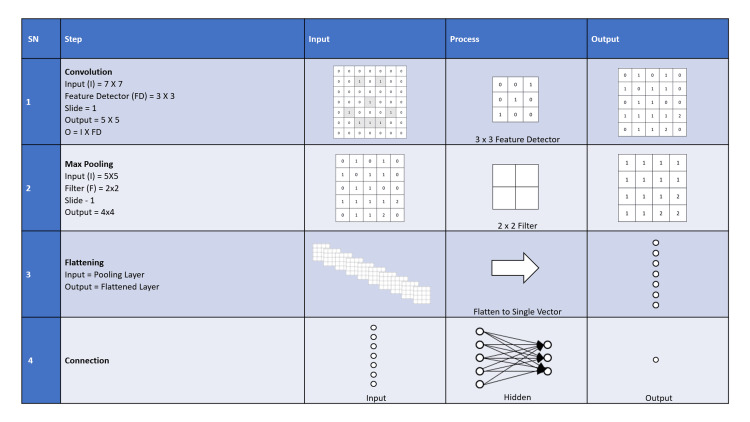
Convolutional neural network pre-processing and modeling. Authors’ own creation [20].

The convolutional neural network learns over time by using weights and biases on the inputs to derive a function [20]. The deviations are fed back so that weights and biases can be adjusted [20,21]. During image processing, a convolutional neural network focuses on reducing an image by keeping the critical feature intact, which is helpful in better prediction. Once learning is achieved, it can be used to analyze new inputs to derive the output of the function. This is similar to biological neural networks [20-26]. Figure 2 explains the convolutional neural network pre-processing and modeling.

Decoding photoreceptors

While decoding and simulating the functionalities of the optic nerve and brain may be too far-fetched now, the next challenge is to crack the functionality of the photoreceptors to understand if there is a correlation between the photons received and the electric signals generated. The hope is to use deep learning techniques to decode this correlation. This can be used to artificially generate the electrical signals from the photons and trigger the optic nerve for people who had normal vision earlier and still have the sensation intact in the optic nerves [20-29].

Electroretinography techniques are used to record the electronic signals generated by photoreceptors in response to light. Electrodes are placed on the surface of the cornea to measure the response from the retina [27]. The electrical signals generated are very small in the range of 10^-6^ to 10^-9^ volts. The simulation performed on normal human eyes show an a-wave (negative deflection) which is followed by a b-wave (positive deflection) [28]. Electroretinography performed on eyes adapted to dark will trigger the rod system whereas that performed on eyes adjusted to light will trigger the cone system [29]. Simulation of input light signals (electromagnetic radiation) and output electrical signal data using electroretinography with normal vision of several individuals needs to be performed [27].

**Figure 3:**
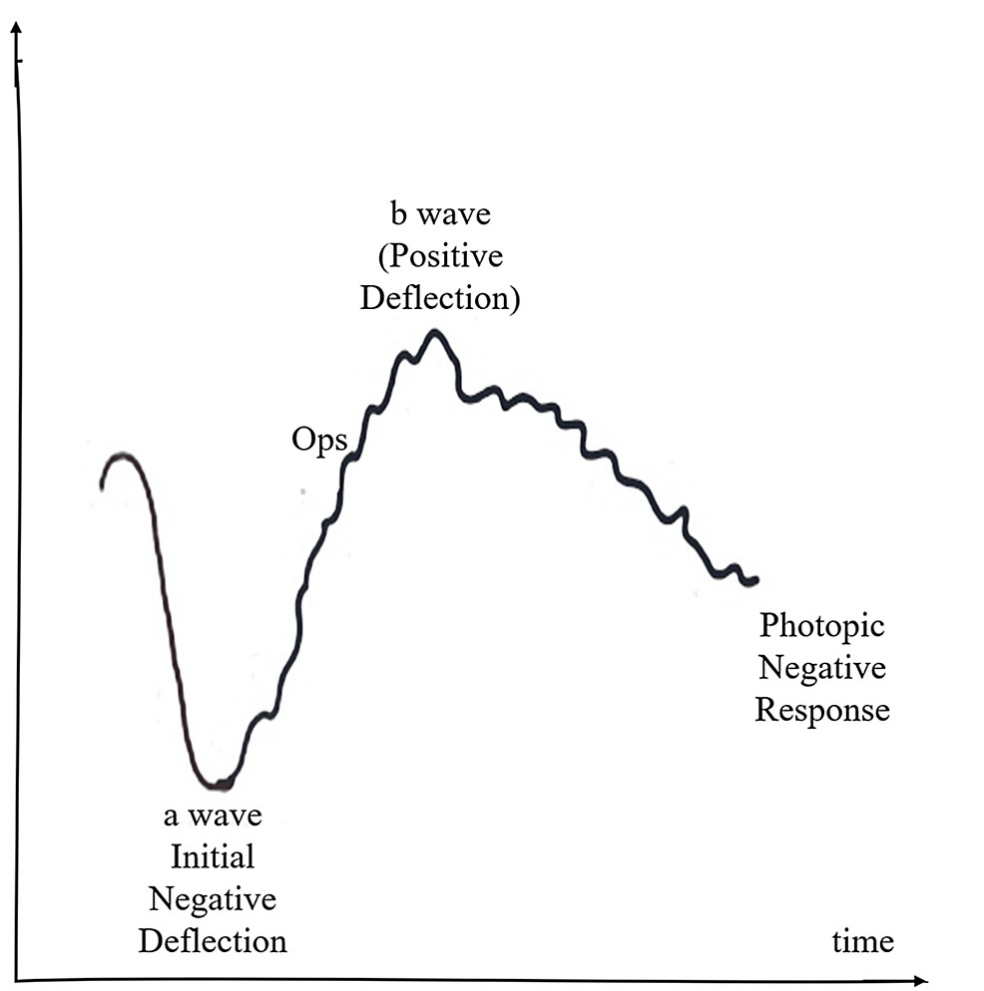
Electroretinography Authors’ own creation [27].

Once we have millions of such records, this could be fed into a convolutional neural network that uses deep learning techniques to find the correlation between the electromagnetic light waves and electronic signals generated in response. This function helps adjust the weights and biases of the parameters to be able to analyze any new scenarios presented to the eye [20-29]. Figure 3 shows the graph for electroretinography.

Let us take the example where a cat’s image is shown to an individual with normal vision. When electroretinography is performed during this process, the complex data correspond to the electrical signals generated, read, and recorded [27,28].

This needs to be repeated with thousands of individuals with normal vision so that the data recorded are substantial and provide sufficient facts to derive convincing results [30]. There may be small variations in the output as we take the readings for different individuals. This would make the system learn better and more robust for the detection of different actual appearances of a cat.

The next step is to create variants of the image. Options could be different cat images, showing a partial image, blurred image (e.g., through the bushes), images with different luminance, the image at different rotational angles, and images with different backgrounds (e.g., residential, grassland, bushes, etc.) [31,32]. We need to record the data corresponding to the electrical signals generated.

Furthermore, an improvement to the input image can be made by feeding a movable image of the cat. For example, approaching the eye, going away from the eye, moving in several directions, including walking, running, jumping, etc. [31,32].

The pooling concept would help to reduce the dimensions of representation and will make the system robust to handle variations and distortions [33]. The algorithm would be insensitive to slight changes in the pixels of the cat’s image. It will also help reduce repeated learning to some extent as minor variations need not be fed to the algorithm [33]. The neural system has analogous behavior with a combination of simple and complex cells. Simple cells arouse responses in each specific spatial location. Complex cells depend on pooling the responses from simple ones [33].

In each such case mentioned above, the feature matrix corresponding to input data is fed to the convolutional neural network which starts with certain initial weights and biases. The actual output is also fed to a convolutional neural network. Consequently, the algorithm learns in a series of feed-forward and backward propagation of data through its layers [20].

Once the algorithm is sufficiently trained, it knows how to detect a cat. At the same time, the system knows what kind of electrical signals are generated for different variants. It has a correlation to the output signal generated when a cat appears.

Encoding photoreceptors

**Figure 4:**
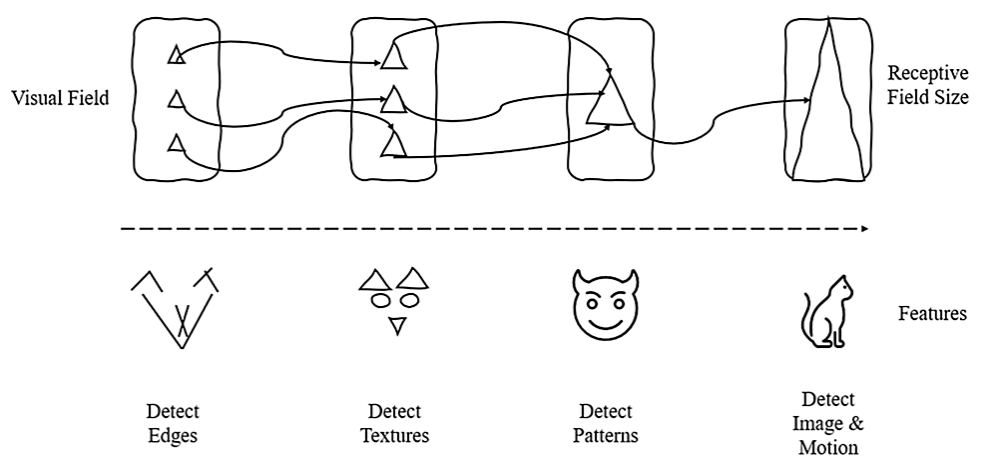
Example of an image detection. Authors’ own creation [31,32].

A highly sophisticated camera could be used to capture the light waves coming from a natural scenario. For ease of usage, a micro camera can be designed in a way that it can also be placed comfortably in subjects where the lens is dysfunctional [10,34]. There could also be the possibility to embed the camera into the eye socket (only in certain subjects where feasible) [34]. The electromagnetic light signals from the camera can be then passed on to a microdevice embedded with the trained convolutional neural network, which would then act like the photoreceptor [10,34]. The whole device can be built as a sophisticated wearable [34]. This can use augmented reality to process images and use photodiodes to generate pulses of electric current. This can help to stimulate the nerve cells in the inner nuclear layer [10,34]. Once the electrical signals are generated, they can be fed to the normal functioning optic nerve which will then pass it to the brain according to its normal course [10,34]. Figure 4 shows an example of image detection.

Going back to the example of the cat. Consider that there is an appearance of a cat out of the bushes and jumping onto a wall. The same image could also be fed to a multilayered convolutional neural network. The convolutional neural network processes the image step by step. At each subsequent layer, more complex features are determined [32,33]. The first layer (group of layers) would engage in detecting edges that are the simplest to figure out. The next layer (group of layers) would use the information coming from previous layers, in this case by combining the information of the edges to detect textures [32,33]. Subsequent layers would combine the textures to derive patterns that can then help to derive parts of the image, e.g., nose, eyes, ear, tail, etc. [32,33]. Further layers would try to assemble the parts derived, check the texture, and determine the movements [32,33]. Consequently, the system would use its learning to assemble the learning parts and determine the possibility of a cat in motion from a lower level to a higher level. It would be based on its learning to generate the required output electric signals which could then be passed on to the optic nerve [10,34].

Variance and complexity

The human eye receives numerous photo signals in a second of different types and complexities. It is necessary to consider these variances during simulation to maximize training and coverage of use cases [35]. Various scenarios need to be considered by altering different parameters such as colored light (in bright daylight, different colors) versus black and white (in dark), stationary objects versus objects in motion [35], objects placed at varying distances, objects moving at different speeds and directions (linear, periodic, oscillatory, circular), object moving toward and away from the eye, different objects with varied textures, distinct images versus blurred images, and parts of objects separated and assembled [35]. A combination of the above variances is also necessary for the simulation to improve the accuracy of the convolutional neural network.

Tests and feedback

The tests with the proposed sophisticated wearable need to be conducted among individuals who have vision impairment due to impacted photoreceptor activity. They need to be exposed to different light signals in natural use cases and interpretations need to be recorded. The deviations observed can act as inputs for further use cases to be considered for training the convolutional neural network with normal vision [36,37]. We need to start with basic use cases revolving around the variances mentioned in the previous section. Slowly, the complexities need to be increased. A series of trials and feedback are necessary to improve the convolutional neural network over time.

## Conclusions

The paper provides a concept of how we can go about using convolutional neural networks to simulate photoreceptor activity. This would help in resolving impaired vision due to pure photoreceptor issues (limited to a class of individuals who had normal vision earlier but got impacted due to photoreceptor inactivity). It explains what could be done to learn from normal photoreceptor activities, and how this learning can be captured in a convolutional neural network and used to treat specific cases of vision impairment. It needs to be checked if there are other parameters that should be considered in combination apart from the electromagnetic signals to better stimulate photoreceptors. The experiments with actual readings are outside the scope of this review.

## References

[1] (2022). Britannica. Human eye. https://www.britannica.com/science/human-eye.

[2] Stone WL, Patel BC, Basit H, Salini B (2022). Retinopathy. https://pubmed.ncbi.nlm.nih.gov/31082175/.

[3] Zang J, Neuhauss SC (2021). Biochemistry and physiology of zebrafish photoreceptors. Pflugers Arch.

[4] Liu Z, Kurokawa K, Zhang F, Lee JJ, Miller DT (2017). Imaging and quantifying ganglion cells and other transparent neurons in the living human retina. Proc Natl Acad Sci U S A.

[5] Banerjee S, Horton J (2021). Lenses and Spectacles to Prevent Myopia Worsening in Children. Canadian Agency.

[6] Zeri F, Durban JJ, Hidalgo F, Gispets J (2010). Attitudes towards contact lenses: a comparative study of teenagers and their parents. Cont Lens Anterior Eye.

[7] Purves D, Augustine GJ, Fitzpatrick D (2001). Phototransduction. Neuroscience. 2nd Edition.

[8] (2022). MSAIL. Do convolutional neural networks mimic the human visual system?. https://MSAIL.github.io/post/cnn_human_visual/.

[9] Nowik K, Langwińska-Wośko E, Skopiński P, Nowik KE, Szaflik JP (2020). Bionic eye review - an update. J Clin Neurosci.

[10] Lewis PM, Ackland HM, Lowery AJ, Rosenfeld JV (2015). Restoration of vision in blind individuals using bionic devices: a review with a focus on cortical visual prostheses. Brain Res.

[11] Naifeh J, Kaufman EJ (2022). Color Vision. https://pubmed.ncbi.nlm.nih.gov/29261952/.

[12] Ritter E, Elgeti M, Bartl FJ (2008). Activity switches of rhodopsin. Photochem Photobiol.

[13] Yoshizawa T (1984). Photophysiological functions of visual pigments. Adv Biophys.

[14] Bliss AF (1946). The chemistry of daylight vision. J Gen Physiol.

[15] Woelders T, Leenheers T, Gordijn MC, Hut RA, Beersma DG, Wams EJ (2018). Melanopsin- and L-cone-induced pupil constriction is inhibited by S- and M-cones in humans. Proc Natl Acad Sci U S A.

[16] Baudin J, Angueyra JM, Sinha R, Rieke F (2019). S-cone photoreceptors in the primate retina are functionally distinct from L and M cones. Elife.

[17] Saari JC (2016). Vitamin A and vision. Subcell Biochem.

[18] Kusakabe TG, Takimoto N, Jin M, Tsuda M (2009). Evolution and the origin of the visual retinoid cycle in vertebrates. Philos Trans R Soc Lond B Biol Sci.

[19] Doly M (1994). Transduction of the light message: from the photon to the optic nerve. Fundam Clin Pharmacol.

[20] Kriegeskorte N, Golan T (2022). Neural network models and deep learning. Curr Biol.

[21] Richards BA, Lillicrap TP, Beaudoin P (2019). A deep learning framework for neuroscience. Nat Neurosci.

[22] Liang X, Xu L, Liu J, Liu Z, Cheng G, Xu J, Liu L (2021). Patch attention layer of embedding handcrafted features in CNN for facial expression recognition. Sensors (Basel).

[23] Zhang Q, Huang N, Yao L, Zhang D, Shan C, Han J (2019). RGB-T salient object detection via fusing multi-level CNN features. IEEE Trans Image Process.

[24] Wang SH, Phillips P, Sui Y, Liu B, Yang M, Cheng H (2018). Classification of Alzheimer's disease based on eight-layer convolutional neural network with leaky rectified linear unit and max pooling. J Med Syst.

[25] Tripp B (2019). Approximating the architecture of visual cortex in a convolutional network. Neural Comput.

[26] Boynton GM (2005). Attention and visual perception. Curr Opin Neurobiol.

[27] Ziv B (1961). Electroretinography. N Engl J Med.

[28] Creel DJ (2019). Electroretinograms. Handb Clin Neurol.

[29] Joachimsthaler A, Kremers J (2019). Mouse cones adapt fast, rods slowly in vivo. Invest Ophthalmol Vis Sci.

[30] Burt R, Thigpen NN, Keil A, Principe JC (2021). Unsupervised foveal vision neural architecture with top-down attention. Neural Netw.

[31] Sun Y, Hu J, Wang W, He M, de With PH (2021). Camera-based discomfort detection using multi-channel attention 3D-CNN for hospitalized infants. Quant Imaging Med Surg.

[32] Sun Y, Shan C, Tan T, Tong T, Wang W, Pourtaherian A, de With PH (2019). Detecting discomfort in infants through facial expressions. Physiol Meas.

[33] Laskar MN, Sanchez Giraldo LG, Schwartz O (2020). Deep neural networks capture texture sensitivity in V2. J Vis.

[34] Mathieson K, Loudin J, Goetz G (2012). Photovoltaic retinal prosthesis with high pixel density. Nat Photonics.

[35] Ahissar E, Arieli A (2012). Seeing via miniature eye movements: a dynamic hypothesis for vision. Front Comput Neurosci.

[36] Stingl K, Bartz-Schmidt KU, Besch D (2012). [What can blind patients see in daily life with the subretinal Alpha IMS implant? Current overview from the clinical trial in Tübingen]. Ophthalmologe.

[37] Stingl K, Bartz-Schmidt KU, Besch D (2015). Subretinal visual implant alpha IMS--clinical trial interim report. Vision Res.

